# The millennial dynamics of malaria in the mediterranean basin: documenting *Plasmodium spp.* on the medieval island of Corsica

**DOI:** 10.3389/fmed.2023.1265964

**Published:** 2023-12-08

**Authors:** Mahmoud Abdelwadoud Boualam, Anne-Gaëlle Corbara, Gérard Aboudharam, Daniel Istria, Michel Signoli, Caroline Costedoat, Michel Drancourt, Bruno Pradines

**Affiliations:** ^1^IHU Méditerranée Infection, Marseille, France; ^2^Aix-Marseille Univ, Institut de recherche pour le développement , Microbes, Evolution, Phylogénie et Infection, IHU Méditerranée Infection, Marseille, France; ^3^Aix-Marseille Univ, Centre national de la recherche scientifique, LA3M, Aix-en-Provence, France; ^4^Aix-Marseille Université, Centre national de la recherche scientifique, Établissement français du sang, Anthropologie bio-culturelle, droit, éthique et santé, Marseille, France; ^5^Unité Parasitologie et Entomologie, Département Microbiologie et Maladies Infectieuses, Institut de Recherche Biomédicale des Armées, Marseille, France; ^6^Aix-Marseille Univ, Institut de recherche pour le développement, Service de Santé des Armées, Assistance publique - Hôpitaux de Marseille, VITROME, Marseille, France; ^7^Centre National de Référence du Paludisme, Marseille, France

**Keywords:** *Plasmodium*, *Plasmodium falciparum*, *Plasmodium ovale*, ancient malaria, Corsica, paleo-auto-immunohistochemistry, metagenomic, immunochromatography

## Abstract

**Introduction:**

The lack of well-preserved material upon which to base the paleo-microbiological detection of *Plasmodium* parasites has prevented extensive documentation of past outbreaks of malaria in Europe. By trapping intact erythrocytes at the time of death, dental pulp has been shown to be a suitable tissue for documenting ancient intraerythrocytic pathogens such as *Plasmodium* parasites.

**Methods:**

Total DNA and proteins extracted from 23 dental pulp specimens collected from individuals exhumed from the 9th to 13th century archaeological site in Mariana, Corsica, were analyzed using open-mind paleo-auto-immunohistochemistry and direct metagenomics, *Plasmodium*-targeting immunochromatography assays. All experiments incorporated appropriate negative controls.

**Results:**

Paleo-auto-immunohistochemistry revealed the presence of parasites *Plasmodium* spp. in the dental pulp of nine teeth. A further immunochromatography assay identified the presence of at least one *Plasmodium* antigen in nine individuals. The nine teeth, for which the PfHRP-2 antigen specific of *P. falciparum* was detected, were also positive using paleo-autoimmunohistochemistry and metagenomics.

**Conclusion:**

Dental pulp erythrocytes proved to be suitable for the direct paleomicrobiology documentation of malaria in nine individuals buried in medieval Corsica, in agreement with historical data. This provides additional information on the millennial dynamics of *Plasmodium* spp. in the Mediterranean basin.

## Introduction

Human malaria, caused by six of the 250 known *Plasmodium* spp. parasites, is transmitted through the bite of *Plasmodium*-infected *Anopheles* female mosquitoes (1). Accordingly, *Plasmodium falciparum* (*P. falciparum*), *Plasmodium vivax* (*P. vivax*), *Plasmodium ovale* (*P. ovale*) *wallikeri, P. ovale curtisi*, *Plasmodium malariae* and *Plasmodium knowlesi* have been documented with variable prevalence in the world’s populations. Malaria continues to be a significant public health issue, causing 241 million new cases and 627,000 deaths worldwide in 2021 (2).

**FIGURE 1:**
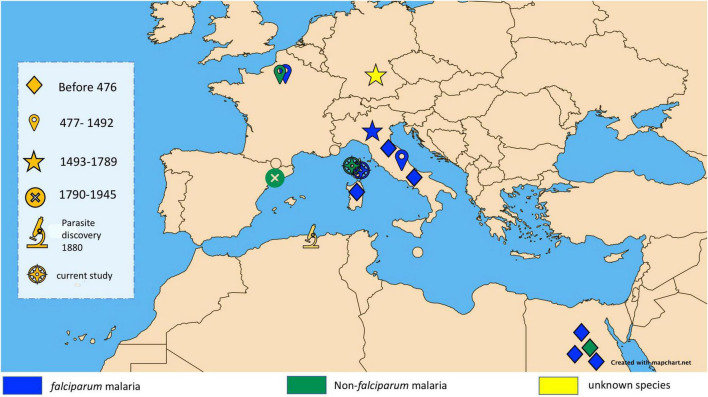
Map showing major ancient malaria diagnoses in the Mediterranean Basin classified by historical period and showing diagnosed species of malaria.

Cases of malaria are largely reported in tropical belt countries, mirroring the geographical distribution of *Plasmodium*-infected *Anopheles* vectors. Europe, where there are no infected vectors, could be regarded as a malaria-free continent. However, a few autochthonous cases have been recorded in Europe over the last two decades, in Corsica in 2006 (3) and in Greece in 2011–2012 (4). These rare contemporary cases provide evidence that Europe was once endemic for malaria, as documented by the paleo-microbiological detection of *Plasmodium* spp. in some individuals exhumed from Italian archaeological sites, including sites in Velia and Vanari, in southern Italy from the 1st and 2nd centuries (5) and the 450 CE Lugnano Teverina site (6), along with diagnosis in the 16th century Medici family in Florence (7). Another case between 1,400 CE and 1,800 CE was also reported in southern Germany (8) (Figure 1). The term “malaria,” derived from the Italian word mal’aria, which literally means “bad air,” links malaria to the moist air around swamps and marshes during the Middle Ages (9, 10).

Corsica is an island which is geographically separated from Italy by less than 90 km and from Sardinia by only 12 km. Historically, it had maritime links with many cities around the Mediterranean Sea. According to ancient texts, it experienced several episodes of malaria during different historical periods (3, 11–14). Miasmas and fevers were reported in the South of Bastia in 1499 (15). Malaria fevers were confirmed during the 17th and 18th centuries. In 1875, 80% of the inhabitants of the eastern coast of Corsica were considered to suffer from malaria due to their sickly pale appearance. Mortality was high but it was impossible to determine the responsibility of malaria among the other fevers such as typhoid. The prisoners of the penal colony of Casabianda near Aleria, opened in 1861, had an annual mortality rate greater than 10%, largely due to malaria, leading to the closure of the penitentiary in 1886 (16). From 1899 to 1903, Laveran confirmed the presence of *Anopheles maculipennis* on the eastern coast where malaria fevers were observed. In 1920, the presence of many cases of falciparum and vivax and some cases of malariae parasites were still reported and associated with plasmodic indices between 20 and 40% in children under 16 years of age (17). In 1946–1947, malaria was still observed in Corsica (18). The campaigns against mosquitoes (larvae and adults), started in 1948, gradually interrupted malaria transmission in Corsica. About 30 autochthonous cases were reported in 1970–1971 (11). An indigenous case of *P. vivax* was described in 2006 (19). Since then, no indigenous malaria transmission has been reported in Corsica.

However, none of these putative episodes observed before the beginning of the 20th century have been confirmed through the direct demonstration of *Plasmodium* spp. Did any cases of malaria exist before the first description of fevers near Bastia in 1499? Paleo-auto-immunohistochemistry, immunochromatographic assays and DNA sequencing are tools that can help us answer this question by detecting parasites, proteins or DNA in dental pulp from teeth collected from human remains buried from the 9th to 13th century.

In this study, we reported the detection of *Plasmodium* spp. in samples collected from a medieval archaeological site in Corsica.

## Materials and methods

### Archaeological site

**FIGURE 2:**
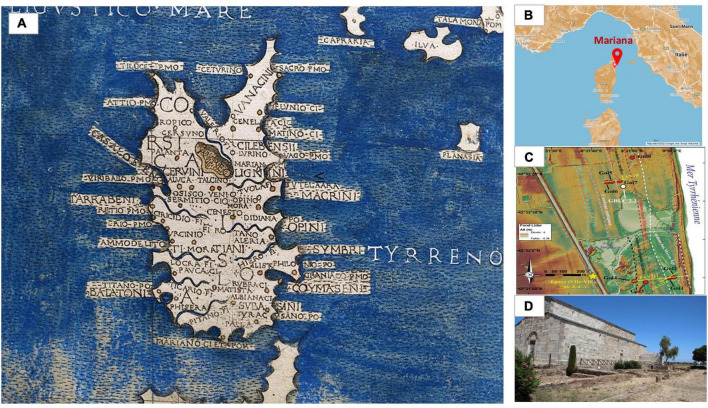
**(A)** Cartography of the island of Corsica in 1482 by Francesco Berlinghieri, including the name and location of the ancient city of Mariana. **(B)** Location of the archaeological site of Mariana in Corsica. **(C)** LIDAR processing of the sectors, showing a progradation of the coastline and the formation of cordons which probably led to the appearance of swamps behind (20). **(D)** Church of the canonica, as the remaining vestige of the city Mariana.

**FIGURE 3:**
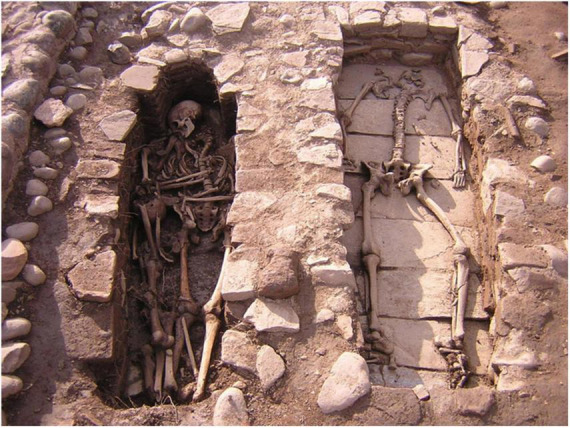
Graves of individuals T 40 and T 41. Photo credits CNRS.

The Roman colony of Mariana, in the north-east of the island of Corsica, was established at the start of the 1st century BCE. It considerably evolved over time with the development of wetlands at the gates of the city, as demonstrated by recent geoarchaeological studies. The coastline, which is now 3 km from Mariana, has been subjected to a 400 m progradation, creating lagoon areas behind the dunes from late Antiquity onward. The Golo River, which now flows south of the site, has moved several times. During the early Middle Ages, one of its arms – if not its main course – ran alongside the southernmost buildings and a quay was built at the eastern end of the settlement. Finally, the Biguglia lake, a 1,450 ha body of brackish water located 3 km to the north, dried up during the 10–12th centuries, causing significant silting (20, 21) (Figure 2). As the only episcopal seat on the island, the city features several Christian buildings of worship. The southern basilica, erected at the beginning of the 5th century, has been restructured a number of times. It served as a cathedral until the construction of a second church, and was consecrated in 1,119 (22). A cemetery developed around the southern building from the late 9th or 10th century. The site was completely abandoned in the second half of the 15th century, in favor of the city of Bastia [(22), p.82 and 188–190]. Eighty-eight tombs, all located in and around the southern border of the church, were excavated in the late 1950s and early 2000s, yielding the archaeological materials investigated here. Tombs 72 and 18 were located in the central nave and the southern aisle, respectively. Tombs 40, 41, 44 were located in the vestibule (Figure 3). These two sectors were occupied by the episcopal palace from the second half of the 12th century [(22), p. 184–187]. All tombs were dug from the floor of the church within a large chronological range between the end of the 9th century and the second half of the 12th century. Tomb 18 was radiocarbon dated to between 1,034 and 1,215 (age calibrated to 2 sigma with 95% probability) (Lyon 2385, Age 14c BP: 900 ± 30) and a date between the second third of the 11th and the middle of the 12th century was accepted. Tombs 31, 34 and 35 were located outside and south of the church, in earth and building materials that were radiocarbon dated to the 10th and 11th centuries (T.17 and T.20) and covered by silt deposited before the 16th century [(22), p. 82]. The endemicity of malaria in this part of Corsica was historically attested at the beginning of the 20th century by a doctor who stated: “*Only the sanitation of the plain which extends from Bastia to the Golo could reduce the frequency and even get rid of the malaria attacks which return periodically*” (23).

### Anthropological investigations

The sex of adults was determined using the probabilistic sex determination (PSD) method based on metric data taken from the coxal bone (24). When this method could not be implemented, methods based on observing the morphological characters of the coxal bone were used (25). To estimate the age at death of immature individuals, different indicators were observed, including dental maturation (26–28). For adolescents, this relied on stages of bone maturation and/or stages of epiphyseal synostosis (29). For young adults, the stage of fusion of the epiphyses and clavicle sternal end were observed (30).

### Dental pulp collection

A total of 23 teeth (one tooth per individual) were selected from a total of 45 teeth taken from 27 different individuals, after discarding teeth in poor condition (Supplementary Table 1). These samples were a gift by the “Laboratoire d’Archéologie Médiévale et Moderne en Méditerranée” (Dr Corbara and Pr Istria) and “Anthropologie bio-culturelle, droit, éthique et santé” (Pr Costedoat and Pr Signoli). All teeth were handled under a hood in a paleomicrobiology laboratory, where no *Plasmodium* had previously been handled. The exterior of the tooth was disinfected using a sterile compress soaked with 100% ethanol followed by 9% w/v sodium hypochlorite (NaClO) to avoid any risk of contamination from the tooth surface. After decontamination, a fracture line was made along the length of the tooth, without extending to the pulp cavity, using a sterile diamond disc and the tooth was fractured into two using a sterile straight osteotome. The dental pulp was then scraped out using a dental excavator into a sterile Eppendorf tube and stored at −20°C until used (31).

A tooth collected from an unrelated archaeological site of Boulogne-sur-Mer (dated from the 18th century) devoid of any historical and anthropological pieces of evidence for malaria was used as negative control.

### Paleoserum extraction

Paleoserum was extracted from the dental pulp as previously described (32). Briefly, remaining 100 μL of supernatant and debris pellet from lysis treatment for aDNA extraction were mixed with 200 μL of ethylenediaminetetraacetic acid (EDTA) was sonicated 5 × 1 min at 6,000 J and incubated overnight at 4°C under agitation. The mixture was then centrifuged for 30 min at 17,000 *g* at room temperature and the supernatant (supernatant no. 1) was stored at −20°C. The remaining pellets were washed twice with 200 μL of sterile distilled water and incubated for 48 h at 75°C in 100 μL of 50 mM ammonium bicarbonate solution, pH 7.4. The supernatant (supernatant no. 2) was then collected by centrifugation at 17,000 *g* for 30 min at room temperature and stored at −20°C. The two supernatants (nos. 1 and 2) were pooled and dialyzed using the Slide-A-Lyzer MINI Dialysis Device, 2 K MWCO (Pierce Biotechnology, Rockford, IL, USA) with one liter of dialysis solution (50 mM ammonium bicarbonate) for 4 h in the first round, and overnight in the second round. The resulting solution contained ancient proteins, including immunoglobulins (paleoserum), and was freeze-dried before being suspended in 30 μL of phosphate-buffered saline (PBS). Protein quantification was performed using a Qubit assay (Thermo Fisher Scientific, Waltham, MA, USA) for each paleoserum.

### Paleo-auto-immunohistochemistry

The dental pulp was scraped with a sterile scalpel into Eppendorf tubes and rehydrated for 24 h at room temperature in 500 μL of 1% formalin 50 vol, 96% ethanol, 30 vol and 5% ammonium bicarbonate, 20 vol, [adapted from Sandison et al. (33)]. Hydrated samples were homogenized and 100 μL of the suspension was cytocentrifuged for 7 min at 800 revolutions per minute (Shandon Cytospin 4 Cytocentrifuge, Thermo Electron Corporation, Waltham, MA, USA), to prepare four slides for each dental pulp sample (Supplementary Figure 1). One positive control slide was incubated with 200 μL of pooled of four leftover serum specimens diluted at 1:500 collected from patients exhibiting positive *Plasmodium* spp. parasitemia, one patient with 1.3% *P. falciparum* parasitemia, one with 0.8% *P. vivax* parasitemia, one with 0.2% *P. malariae* parasitemia, and one with 0.04% *P. ovale* parasitemia. A negative control slide was incubated with 200 μL leftover serum sample collected from a malaria-free patient at 1:500 dilution. A third slide was incubated with 200 μL paleoserum collected from the same individual as the investigated dental pulp at 1:20 dilution. A fourth slide was observed using electron microscopy (Supplementary Figure 1). The slides were incubated for 15 min with a permeabilization buffer containing 10% foetal bovine serum (FBS), 0.1% Triton X-100 and 0.01% saponin in PBS, washed three times with PBS, and incubated for 30 min with blocking buffer (1 XPBS, 10% FBS) followed by a PBS wash. One slide was then incubated overnight with a positive control serum mix diluted in 5% FBS-PBS at 1:500, a second slide with a malaria-free negative control serum diluted in 5% FBS-PBS at 1:500, and a third slide incubated with homologous paleoserum diluted in 5% FBS-PBS at 1:20. The slides were washed twice with 10% FBS-PBS for 5 min and incubated with secondary anti-human IgG Alexa-647 (Sigma-Aldrich, Saint-Quentin-Fallavier, France) diluted at 1:100 in 5% FBS-PBS and Phalloïdin Alexa-448 (Biotium, Fremont, CA, USA), diluted at 1:250 in 5% FBS-PBS for 1 h, and washed three times with PBS. After incubation at 20°C in a wet chamber, the slides were stained with Hoechst 3342 (Ref. 62249, Thermo Fisher Scientific) at 1:500 dilution in H_2_O for 30 min to reveal nuclei before a final wash with sterile water. The slides were observed and analyzed by confocal laser scanning microscopy (Zeiss LSM800, Zeiss, Jena, Germany) using 405 nm, 488 nm, and 640 nm lasers to reveal Hoechst-3342-stained nuclei, Alexa-448 and Alexa-647 stained actin and anti-human IgG, respectively. An additional 561-nm laser observation was made to control for non-specific fluorescence.

### Immunochromatographic assay

Immunochromatographic assays detected the PfHRP-2 (histidine rich protein-2) antigen specific to *P. falciparum*, the pLDH (lactate dehydrogenase) antigen specific to *P. vivax*, and the PAN-pLDH antigen common to all *Plasmodium* species (pan species) (ACRO Biotech, Inc., Malaria, Montclair, CA, USA). Strips were extracted for quantitative reading using the FUSION FX chemiluminescent imaging system and the ImageQuant TL software (Witec AG, Sursee, Switzerland).

### aDNA extraction

aDNA was extracted from ancient dental pulp along with two negative controls, one experimental negative control consisting in PBS in each batch, and one biological negative control consisting in one tooth from the 18th century. The aDNA extraction used in this study was previously described (34). In brief, 1 μL of TISS which is a synthetic extraction control (35) was added to each sample as an external extraction control. The dental pulp was resuspended in 1 mL of lysis buffer (0.45 M EDTA, pH 8.0 mixed with 0.25 mg/mL proteinase K in nuclease-free water) and incubated at 37°C for 16 h using a thermomixer (500 rpm). After 2 min of centrifugation at 16,000 *g*, 900 μL of supernatant was transferred into a 50 mL Falcon tube. Then, 7 mL of freshly prepared binding buffer (5 M guanidine hydrochloride), 40% (vol/vol) isopropanol, 0.05% Tween-20 and 90 mM sodium acetate (pH 5.2)) were added before vortexing. The remaining 100 μL was stored with the pulp debris to be used for further protein extraction, as above. A Qiagen MinElute Silica Spin column (QIAGEN, Hilden, Germany) was placed in a vacuum system to which 700 μL of mixture was added. The same volume was added every time the tube emptied, until the full 7 mL of sample had been adsorbed on the column. To remove the rest of the liquid, a 1 min 16,000 g centrifugation was carried out. After that, 700 μL of wash buffer (PE buffer, QIAGEN, Hilden, Germany) was added to the MinElute column to be washed following a 1 min 5,300 *g* centrifugation, and the contents of the collection tube were discarded. An additional 2-min 16,000 *g* centrifugation was performed to remove any trace of washing buffer. The column was placed into a sterile 1.5 mL LoBind Eppendorf tube and 12.5 μL of pre-warmed nuclease-free water was added directly to the membrane of the column, followed by 1 min of centrifugation at 16,000 *g* to elute DNA. The last step was repeated a second time, resulting in a final 25 μL volume of purified aDNA.

### Metagenomic analysis

A first sequencing run incorporated pooled aDNA from the 23 teeth, normalized using the Qubit dsDNA High Sensitivity Assay Kit (Life Technologies, Villebon-sur-Yvette, France) and a dilution was performed to obtain 1 ng/uL. Sequencing was performed following the Illumina Nextera-XT paired-end protocol on the MiSeq instruments (Illumina Inc., San Diego, CA, USA). After tagmentation, a limited 12-cycle PCR amplification was performed to complete tag adapters and introduce dual-index barcodes. Genomic aDNA was then purified on AMPure XP beads (Beckman Coulter Inc., Fullerton, CA, USA). In addition, the libraries were normalized manually. We then pooled all the libraries into one for aDNA sequencing on MiSeq. The size of the library was determined using a 2100 Bioanalyzer and a High Sensitivity DNA Kit (Agilent, Santa Clara, CA, USA) and the concentration was determined by a Qubit. A sequencing blank was carried out throughout the preparation of the library as negative controls. A second sequencing run incorporated a selection of 14 individual aDNAs for NovaSeq™ 6000 (Illumina Inc.) sequencing, after a library was prepared as mentioned above. Library pooling (3 nM) and denaturation were performed following standard protocol A from the NovaSeq 6000 System Denature and Dilute Libraries Guide (document #1000000106351 v03), with a 15-h sequencing run performed with a NovaSeq 6000 SP Reagent Kit (100 cycles) v1.5 (Illumina, Inc.) Pre-processing sequencing data was decontaminated using BBduk tools from Galaxy Europe online software (Galaxy)^1^ by removing the sequencing blank data, after adapter trimming using CLC Genomics Workbench. Taxonomic assignment was then performed using Kraken2, which allowed for the rapid taxonomic classification of metagenomic data based on the k-mer approach, with standard database plus protozoa and fungi 2021-05 (36). Kraken 2 results were then converted on Galaxy Europe with convert kraken data tools to translate the results of the Kraken metagenomic classifier to the full representation of NCBI taxonomy. Results were visualized with the Krona pie chart from the converted Kraken2 data. Data generated from the sequencing blank were analyzed using the same approach as a control, except they were decontaminated using BBduk tools. The same process was carried out with metagenomic datasets assembled with metaSPAdes on Galaxy Europe online software. We further investigated reads generated from NovaSeq™ 6000 instruments using a mapping approach against three reference genomes, *P. falciparum*, *P. vivax* and *P. malariae*, using CLC Genomics Workbench software (version 7.5) incorporating a 0.95 similarity fraction, a 0.8 length fraction, a deletion cost of 3, an insertion cost of 3, and a mismatch cost of 2. Mapped reads were then generated and blasted against the NCBI database to confirm their identity. The resulting reads were then decontaminated using BBduk tools from Galaxy Europe online software (Galaxy, see text footnote 1) by removing the blank sequencing data. The data has been deposited and is available on the European Nucleotide Archive website under the accession number PRJEB63329.

## Results

### Anthropology and paleopathology

**FIGURE 4:**
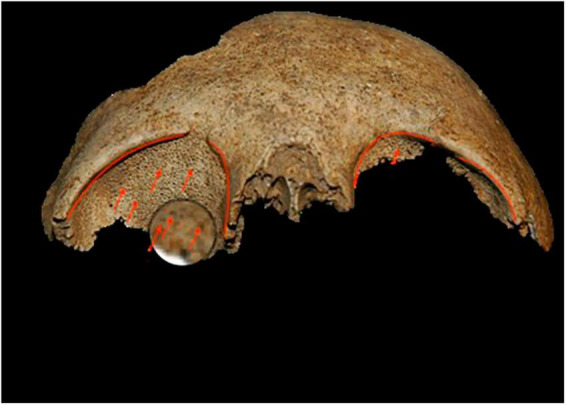
Example of cribra orbitalia observed on individuals at the Mariana site. The photo shows a bilateral moderate-to-severe Cribra orbitalia of individual T 29. Photo credits A-GC.

In Mariana, the individuals who tested positive for malaria died between the end of the 9th century and the second half of the 12th century (Supplementary Table 1). On this site, the funerary practices carried out in relation to the positive test subjects were globally similar, with individual primary deposits for the most part in simple pits. Some tombs stand out (T40, T41, T70) (Figure 3), both for their architecture and their use of masonry chests, as well as the fact that they were reused to bury a second individual or even several successive individuals. The position of these tombs in the internal sectors of the cathedral suggested that they housed the remains of people of privileged status. This observation was reinforced by the presence of clothing and finery in some tombs, denoting high social status. For example, in tomb T18, gold filaments associated with a bone pin were found on the skull of a young woman, and were probably part of a headdress (22, 37). In these skeletons, a 47% prevalence of non-specific inflammatory lesions raised the hypothesis of a systemic, chronic infection in these individuals. Periostitis lesions were observed in nine individuals and cribra orbitalia were noted for four individuals (Figure 4; Supplementary Table 1). Given the position of the Mariana site, located in the heart of a vast marshy area, malaria was suspected, especially since it has long been endemic in Corsica (37).

### Paleo-auto-immunohistochemistry

**FIGURE 5:**
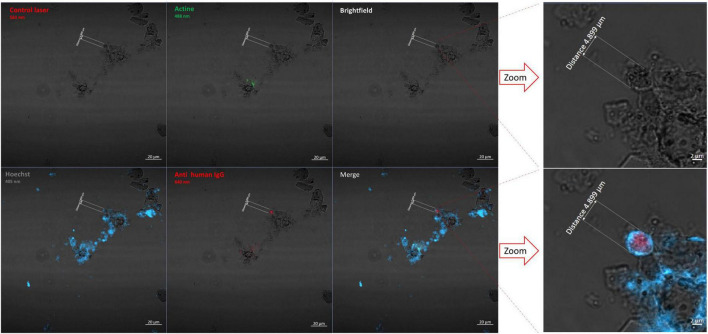
Paleo-autoimmunohistochemistry of individual 18 slide incubated with paleoserum and observed by Confocal Laser Scanning Microscopy using four wavelength lasers (405 nm, 448 nm, 561 nm, and 640 nm) and the brightfield. In red is the anti-*Plasmodium* antibody, in blue the nuclei stained using Hoechst 3342, and in green the Phalloidin stained actin. The merged picture combines all wave acquisitions adding Brightfield. Microscopic observation was conducted expecting to visualize erythrocyte structures, including clusters of erythrocytes caused by dehydration. In the image, a structure corresponding to the size of an erythrocyte can be seen. The scale bar indicates 4,899 μm.

Laser scanning microscopy showed that slides which had been incubated with homologous (from the same individual) paleoserum yielded red after 640-nm laser excitation, consistent with pathogen antigen detection, blue after 405-nm laser, consistent with DNA detection, and green after 488-nm laser excitation, consistent with actin detection (Figure 5). The same observation was made with positive sample slides incubated with a positive control serum reacting with four *Plasmodium* species. Similarly, positive samples incubated with the paleoserum negative control showed no antibody signals in red and revealed only the presence of nuclei in blue and actin in green. Therefore, the paleo-auto-immunohistochemistry results were strongly indicative of the presence of *Plasmodium* antigens in Mariana dental pulp specimens (Table 1; Supplementary Table 1; Supplementary Figure 2). There was no presence of *Plasmodium* antigens in the negative control tooth.

**TABLE 1:** Characteristics of the 23 samples and comparative results of paleo-autoimmunohistochemistry approach, immunochromatographic assay, and metagenomics analyses, *Plasmodium spp*. reads assignation by Kraken2.

Sample ID	Tooth Condition	Estimated age	Sex	Immunochromatographic assay			PAIHC	Metagenomic *Plasmodium Spp*.
				pan-PLDH	PvLDH	PfHRP-2		
T4	Good	20–49 years	M	–	–	–	–	UD
T9	Good	20–49 years	M	–	–	+	+	+
T7	Good	15–19 years	UD	–	–	–	–	+
T6	Good	UD	M	–	–	–	–	UD
T16	Good	>30 years	M	–	–	–	–	+
T18	Good	20–49 years	F	+	+	+	+	+
T22	Good	>30 years	F	–	–	–	–	UD
T8	Good	30–59 years	M	–	–	–	–	UD
T34	Good	UD	UD	–	–	+	+	+
T31	Good	5–9 years/10–14 years	UD	+	–	+	+	+
T36	Good	15–19 years	UD	–	–	–	–	+
T35	Good	30–59 years	M	–	–	+	+	+
T29	Good	5–9 years/10–14 years	UD	–	–	–	–	UD
T48	Good	UD	UD	–	–	–	–	UD
T47	Good	UD	UD	–	–	-	± *	UD
T41	Good	20–29 years	M	–	–	+	+	+
T40	Good	30–59 years	M	–	–	+	+	+
T43	Good	50 years	F	–	–	-	+	+
T44	Good	20–29 years	UD	+	–	+	+	+
T42	Good	30–49 years	M	–	–	–	± *	UD
T70	Good	10–14 years	UD	–	–	–	± *	UD
T72	Good	>60 years	M	–	–	+	+	+
T76	Good	20–49 years	M	–	–	–	–	+
Tooth control	Good			–	–	–	–	–

M = male; F = female; UD = undetermined; PAIHC = Paleo-autoimmunohistochemistry; * positive sample with only paleoserum and not with *Plasmodium spp.* serum.

### Immunodetection of *Plasmodium* antigens

**FIGURE 6:**
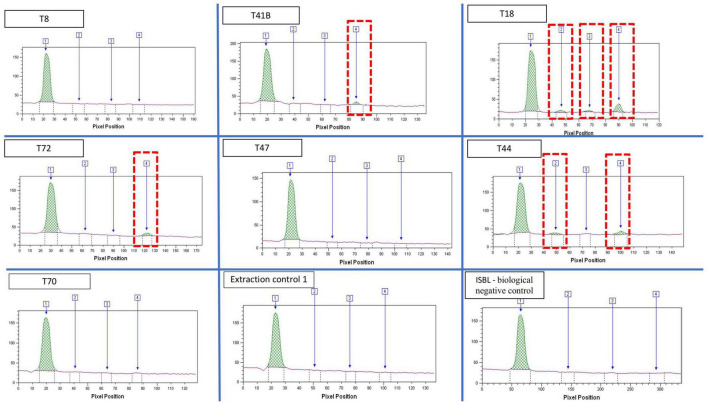
Quantitative analysis of bands extracted from the immunochromatographic assay, imaging and reading using the FUSION FX, band1: internal control; Band2: PAN-pLDH malaria pan species antigen; band 3: pvLDH *P. vivax* antigen; band 4: pfHRP2 *P. falciparum* antigen.

Immunochromatographic assays detected *Plasmodium* spp. antigens in nine of the 23 individuals (39%) (Table 1; Figure 6). The three Pan-pLDH, PvLDH and PfHRP2 antigens were detected in individual T18, Pan-pLDH and PfHRP2 antigens were detected in individuals T31 and T44, and the PfHRP2 antigen was detected in individuals T9, T34, T35, T40, T41, T72, with all the negative controls remaining negative. Nine individuals were positive for the *P. falciparum* antigen and only three were positive for Pan-pLDH. This could indicate an active *P. falciparum* infection at the time of death of the individuals (38). Only one individual was positive for the *P. vivax* “PvLDH” antigen and only three individuals for the pan *Plasmodium* “Pan-pLDH” antigen. This could represent a period of replacement of non-falciparum malaria by *P. falciparum* malaria in this part of southern Europe. The antigenic detection for the tooth control from the 18th century was negative.

### Metagenomics

**FIGURE 7:**
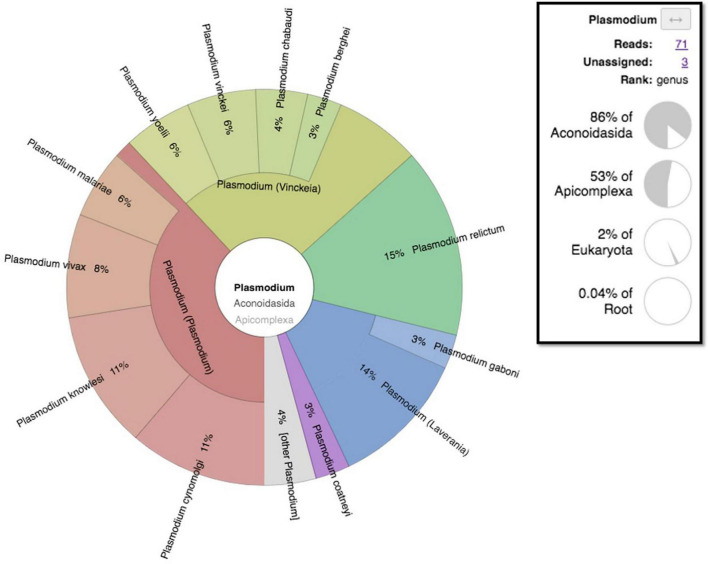
Metagenomic taxonomic assignment pie chart of Mariana sample-generated sequencing reads showing *Plasmodium* assigned reads visualized by the Krona pie-chart in Galaxy Europe.

While both the experimental negative control (PBS) and the biological negative control (dental pulp) yielded no reads assignable to *Plasmodium* spp. (Supplementary Figure 3), a total of 71 reads assigned to *Plasmodium* spp. (Figure 7) were found in pooled dental pulps, while blank sequencing analyzed in parallel to the same sequencing run, did not give any reads assigned to the genus *Plasmodium*. A taxonomic overview of Novaseq sequencing reads assigned a majority of reads to bacteria (90–96%) while only 0.3–3% of reads were eukaryotic. Metagenomic sequencing results indicated the presence of *Plasmodium* assigned reads; however, the very low number of reads meant that the thorough identification and the exact assignment of the *Plasmodium* species were not possible, with reads assigned to *Plasmodium* spp. being different per sample, ranging from 12 to 176 reads. Regarding the classification of data after assembly by MetaSPades, Kraken2 taxonomic classification of the assembled runs after decontamination demonstrated a very low taxonomic assignment to *Plasmodium* sp., ranging from one to four, with a maximum of eight assignments for sample T8 and no assignments for two samples, T35 and T18. Mapping against reference genomes resulted a number of reads between the samples and ranged from 5,450 to 208,185 reads (Supplementary Table 2). After decontamination, only 0.26 to 1.28% of mapped reads remained. To obtain the taxonomic assignation of the remaining mapped reads, we performed at first taxonomic classification using Kraken2, followed by a manual Blastn on NCBI with the standard databases of *Plasmodium* spp. assigned reads. Only three individuals showed indications for *Plasmodium* DNA after a manual Blastn check, while most mapped reads were taxonomically assigned to humans (Supplementary Table 2).

## Discussion

We gathered several lines of evidence for the presence of *Plasmodium* parasites in 9th to 13th populations residing in Corsica, an island in the Mediterranean Sea close to western Italy. First, periostitis lesions were observed in nine individuals and cribra orbitalia were noted for four individuals. Cribra orbitalia and other porotic lesions were more prevalent in areas where the presence of *P. vivax* malaria was observed (39–41). Yet, the cribous lesions were more common in younger individuals (42). However, macroscopic analysis of porotic lesions presents several limitations. These lesions are a consequence of hemolytic anemia as a result of the widespread destruction of erythrocytes during active malaria infection. Other pathologies (iron or nutritional deficiencies) and gastro-intestinal parasitic infections can be the cause of anemia and similar skeletal lesions (43). Malaria is an important contributor to the presence of these lesions; however, porotic lesions are not specific for malaria.

In this study, all negative controls introduced into every experimental procedure were negative, combined with the fact that the ancient specimens were manipulated in a dedicated hood in a laboratory where *Plasmodium* parasites had never previously been handled. Accordingly, both *Plasmodium* antigens and DNA sequences were detected (Table 1), confirming the conclusion that intact parasites once infected dental pulp specimens, equivalent to blood specimens (44, 45). In this study, the pioneering use of Novaseq technology for ancient malaria was challenging. The contamination of published *Plasmodium* spp. genomes, as in the case of *P. vivax* genome known to contain human and bacterial background contamination (46), was a great limitation. The fact that reference databases were incomplete further raised the risk of misreading bioinformatic information, and both false positive and false negative results, due to significant masking as a result of contamination, as is the case with *Plasmodium gallinaceum* 8A (62% masked), *P. falciparum* IT (57% masked), *Plasmodium reichenowi* CDC (55% masked), in which the masked pseudo-reads were identified as corresponding to their host of origin (46). The aDNA quality and the low quantity of generated reads from ancient samples add a further limitation for the diagnosis of malaria by metagenomics. These results support the conclusion reported by Loufouma et al. (45), due to the difficulty of retrieving DNA-based *Plasmodium* indications, making it necessary to use other complementary diagnostic means, as used in this work, such as the detection of specific antigens and paleo-autoimmunohistochemistry.

The nine teeth for which the PfHRP-2 antigen specific of *P. falciparum* was detected were also positive using paleo-autoimmunohistochemistry and metagenomics (Table 1). Immunochromatographic tests and, more particularly, RDTs were showed to be a suitable tool for detecting *Plasmodium* spp. antigens in ancient dental pulp. Immunodetection proved to be a fast, sensitive and very useful technique for screening ancient samples for infectious disease, for the purposes of carrying out an initial diagnosis directly on the excavation sites.

Nevertheless, the combined observations lend paleomicrobiological support to historical reports of febrile conditions compatible with malaria in Corsica in the past, along with attempts at sanitation, as in Biguglia, to the south of Bastia around the turn of the 15th century (14, 15). In the 16th century, the historian Filipini testified to high mortality in the plain of Mariana (14), which had been deserted much like other Corsican coastal plains after repeated Barbarian invasions, forming marshes. This was also documented in the plain of Aléria during the 13th century (13). During the 15th century, the historian Giovanni Della Grossa referred to the “Moscone de Freta,” a fly as big as an ox which poisoned people, suggesting the appearance of malaria. Other testimonies dating from the 18th century indicated bad air and intermittent fevers (13). After the political attachment of Corsica to France in 1768, sanitation in Corsica was still matter of debate in the court of the French kings (14). In 1875, based on the inhabitants appearing to be pale in the face, 80% of the inhabitants of the eastern plain were assumed to have malaria, with high mortality rates, such as in Aléria, where life expectancy was only 24 years (15).

Our observations antedate the antiquity of microbiologically proven malaria in Corsica by 1,600 years, as the only previous documentation was the 1899 microscopic observation of *Plasmodium* parasites, 19 years after their discovery by Nobel prize winner Laveran in 1880 (47). Interestingly, the Mariana site investigated here is close to Italy, where a few paleomicrobiology investigations documented *P. falciparum* in infected individuals from the 1st, 2nd, and 5th centuries (5, 6, 8), in addition to *P. falciparum* retrieved from some 16th century Medici family members in Florence (7) (Figure 1). In addition to previously reported reports of *Plasmodium* spp. around the Mediterranean Basin and beyond, our observations help to capture the millennial dynamics of malaria in this part of the world, with an expanding *P. falciparum* originating from Africa encountering non-*falciparum Plasmodium* species already present in continental Europe (48). Accordingly, specific malarial resistance genetic traits such as glucose-6-phosphate dehydrogenase gene mutations, hemoglobin subunit beta gene mutations, and Duffy blood group mutations were not detected in 224 human European genomes dating from the Upper Paleolithic to Roman periods. This observation suggests a low selective pressure of *Plasmodium* spp. during this period on the European populations, such as that expected for non-*falciparum Plasmodium* parasites (49).

## Conclusion

Combining ancient proteins and aDNA detection and specification as here, partially neutralized limitations in the reduced specificity and sensitivity of any test applied to ancient materials, for the detection of *Plasmodium* in ancient specimens. Indeed, direct next-generation sequencing proved experimentally difficult, fortunately new metagenomic analysis approaches detecting *Plasmodium* signatures using taxonomic assignment approaches proved useful. Interestingly, immunochromatographic tests (more specifically RDTs) here showed to be suitable for detecting *Plasmodium* spp. antigens, are portable techniques opening the possibility for malaria screening and selection of specimens amenable to further in-laboratory analysis in the anthropological scene, an approach we called AnthropoPOC^®^.

Applying methods such as the ones here described to anthropological materials collected in other archaeological sites in the Mediterranean Basin and elsewhere in Europe may contribute to depicter the millennial dynamics of this deadly infection through collaborative works implying historians, anthropologists, ecologists and parasitologists.

## Data availability statement

The data presented in the study are deposited in the European Nucleotide Archive website of EBI repository, accession number PRJEB63329.

## Ethics statement

The studies were conducted in accordance with the local legislation and institutional requirements. The human dental pulp samples used in this study were from gifted from another research group and therefore, ethical approval was not required. The studies involving the use of human blood samples were approved by the “Internal Ethics Committee of the University-Hospital Institute Méditerranée Infection” (Application number 2022-024). Written informed consent to participate in this study was not required from the participants or the participants’ legal guardians/next of kin in accordance with the national legislation and the institutional requirements.

## Author contributions

MB: Data curation, Formal analysis, Investigation, Methodology, Resources, Software, Validation, Visualization, Writing—original draft, Writing—review and editing. A-GC: Resources, Writing—review and editing. GA: Conceptualization, Methodology, Validation, Writing—review and editing. DI: Resources, Writing—review and editing. MS: Resources, Writing—review and editing. CC: Resources, Writing—review and editing. MD: Conceptualization, Formal analysis, Funding acquisition, Investigation, Methodology, Project administration, Resources, Supervision, Validation, Writing—original draft, Writing—review and editing. BP: Conceptualization, Formal analysis, Funding acquisition, Investigation, Methodology, Project administration, Resources, Supervision, Validation, Writing—original draft, Writing—review and editing.

## Footnotes

1
https://usegalaxy.eu/
