# Supplementary material for: The millennial dynamics of malaria in the mediterranean basin: documenting Plasmodium spp. on the medieval island of Corsica
Source: Front Med (Lausanne). 2023 Dec 8;10:1265964. doi: 10.3389/fmed.2023.1265964 (PMC10739463; doi:10.3389/fmed.2023.1265964)
Supplement: Supplementary file 1 [file Data_Sheet_1.PDF]

Supplementary Material

The millennial dynamics of malaria in the Mediterranean basin: documenting *Plasmodium* spp. on the medieval island of Corsica.

Mahmoud A. Boualam[1,2], Anne-Gaëlle Corbara[3], Gérard Aboudharam[1,2], Daniel Istria[3], Michel Signoli[4], Caroline Costedoat[4], Michel Drancourt[1,2], Bruno Pradines[1,5,6,7].

***Correspondence:** Bruno Pradines, Unité parasitologie et entomologie, Département microbiologie et maladies infectieuses, Institut de recherche biomédicale des armées, IHU Méditerranée Infection, 19–21 Boulevard Jean Moulin, 13005 Marseille, France. E-mail address: bruno.pradines@gmail.com

Supplementary Figure 1: Paleo-autoimmunohistochemistry workflow.

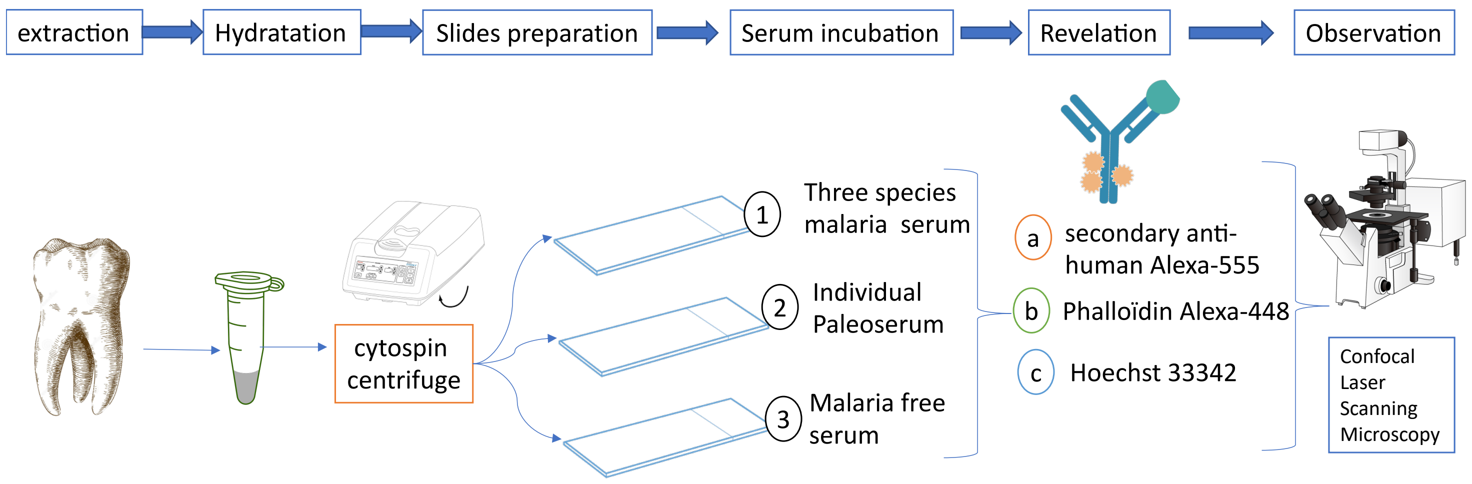

Supplementary Figure 2: Confocal Laser Scanning Microscopy acquisition using four wavelength lasers (405 nm, 448 nm, 561 nm and 640 nm) and the brightfield. In red anti-*Plasmodium* antibody. in blue nuclei stained using Hoechst 3342 and Phalloidin stained actin in green. Merge picture combined all waves acquisition adding Brightfield.

(A): Paleo-autoimmunohistochemistry staining on diagnosed positive *Plasmodium* signal.

(B): Positive serum control using positive serum.

(C): Negative control using negative paleoserum from external anthropological site.

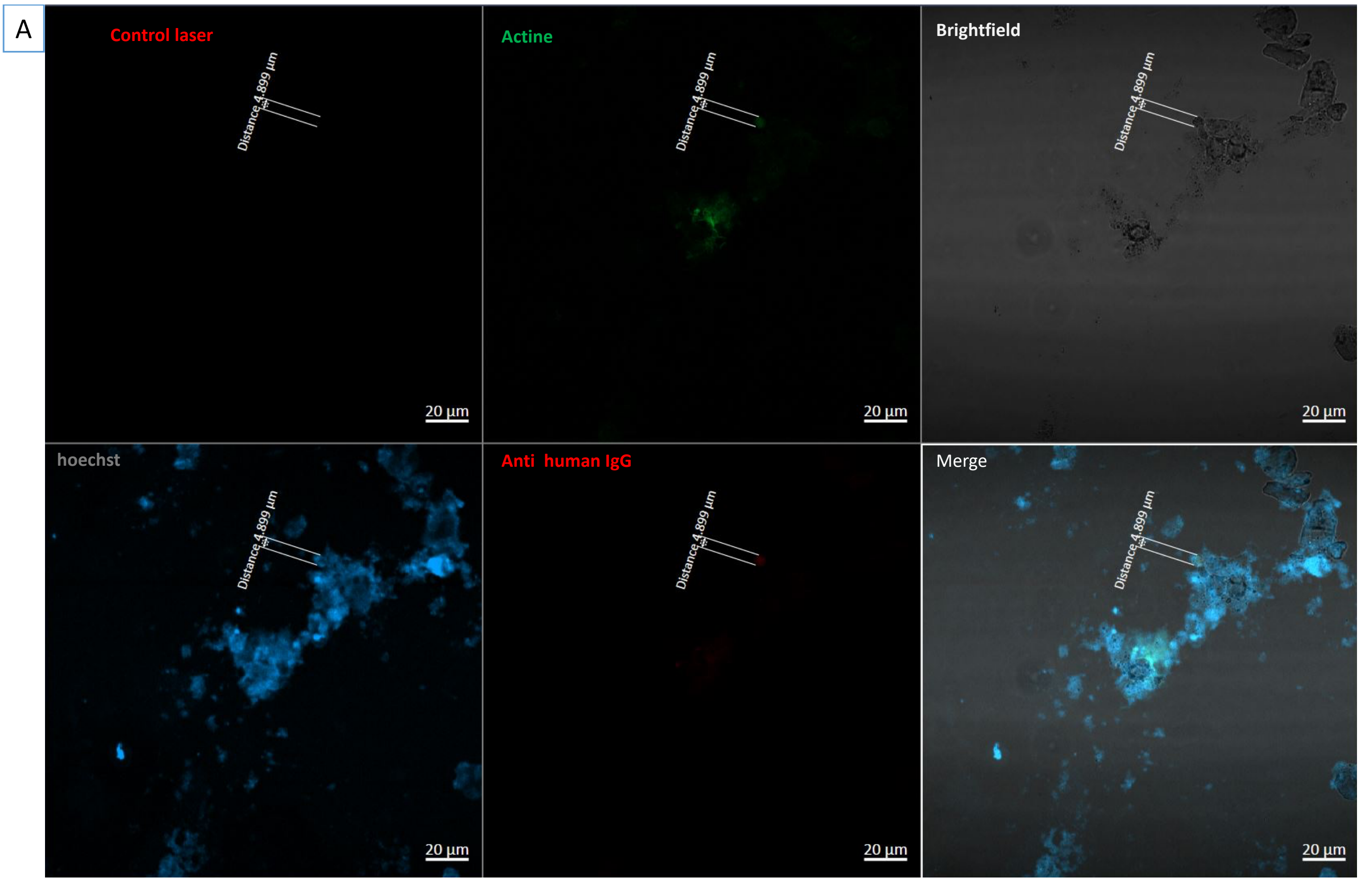

B

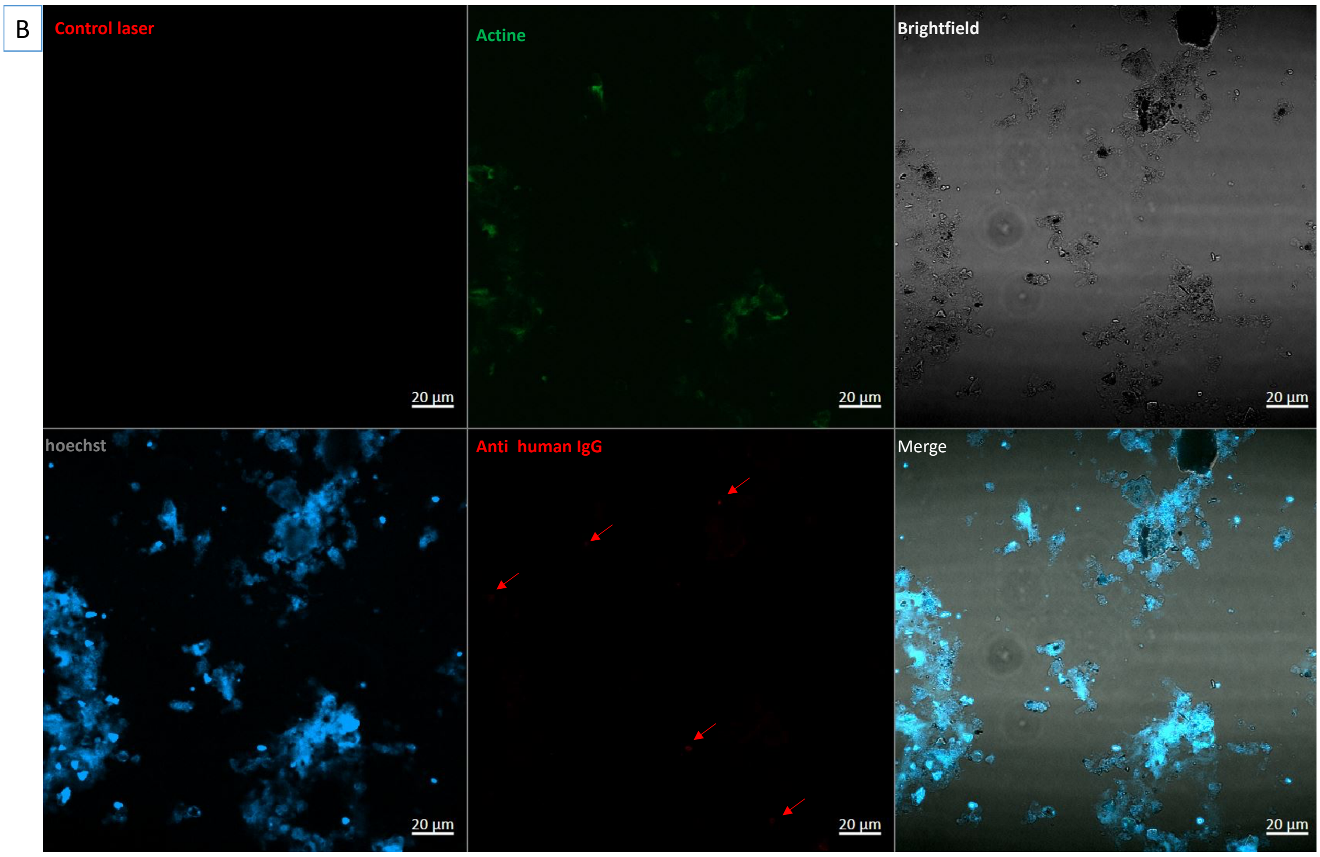
Control laser
Actine
Brightfield
hoechst
Anti human IgG
Merge
20 µm

C

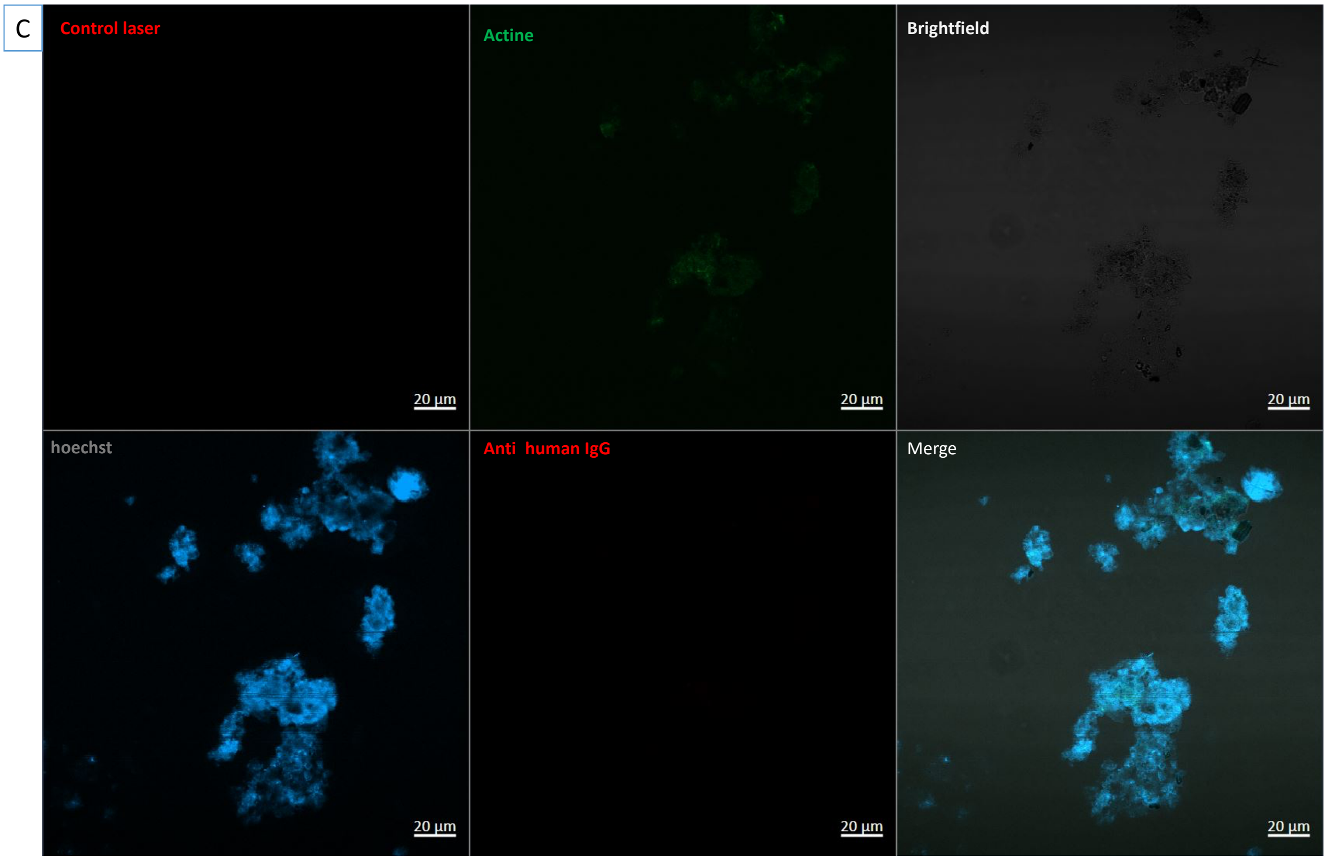
Control laser
Actine
Brightfield
hoechst
Anti human IgG
Merge
20 µm

Supplementary Figure 3

metagenomic taxonomic assignment Pie chart of Mariana samples generated sequencing reads showing Plasmodium assigned reads visualized by Krona Pie-chart in Europa galaxy.

(A): complete taxonomic classification of mariana pool root in the left pie-chart and assigned *Plasmodium* spp. reads in the right pie-chart.

(B): Taxonomic classification of the biological negative control sample Boulogne sur Mer tooth results were showing no read assigned to *Plasmodium* spp.

(C): showing the taxonomic classification of the blank root run in parallel in the same experiment, no read was assigned to *Plasmodium* spp. or even to Eukaryota.

A

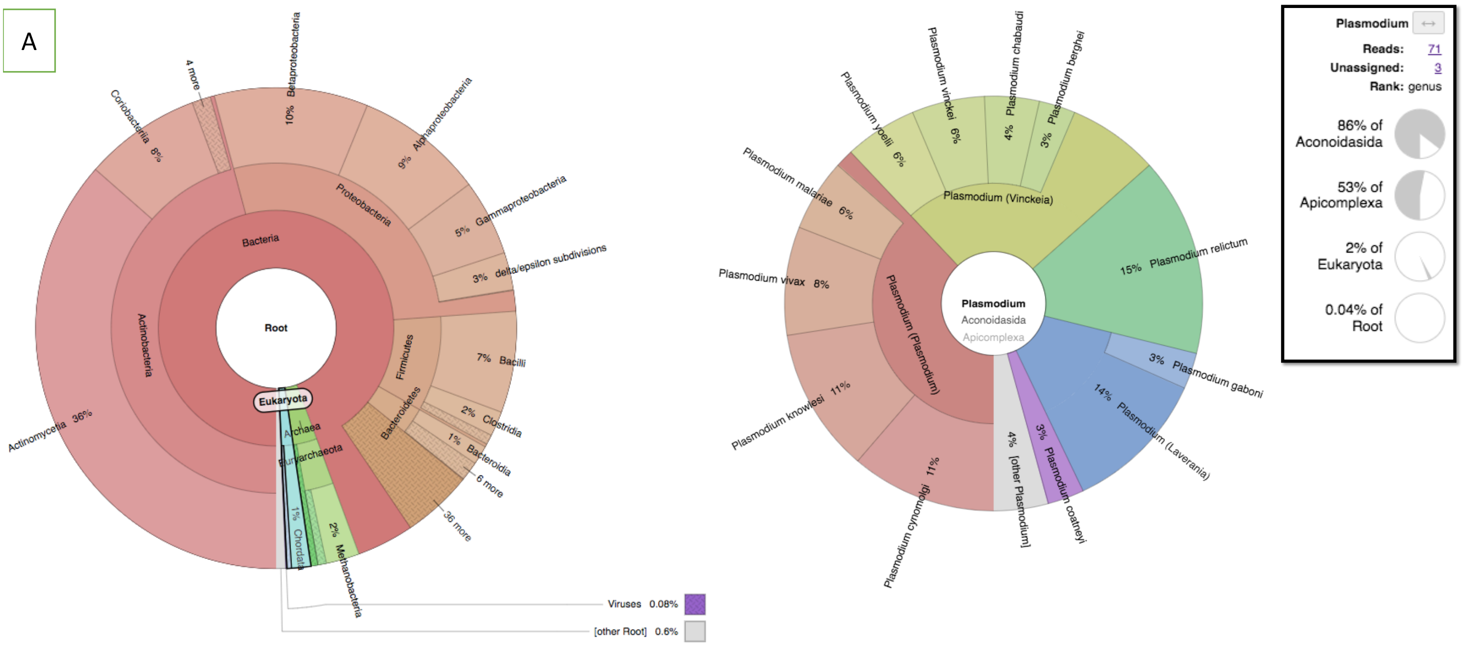

Root
Bacteria
Eukaryota
Archaea
Euryarchaeota
Actinobacteria
Actinomycetia 36%
Coriobacteriia 8%
4 more
10% Betaproteobacteria
9% Alphaproteobacteria
Proteobacteria
5% Gammaproteobacteria
3% delta/epsilon subdivisions
Firmicutes
7% Bacilli
2% Clostridia
Bacteroidetes
1% Bacteroidia
6 more
36 more
1% Chordata
2% Methanobacteria
Viruses 0.08%
[other Root] 0.6%
Plasmodium
Aconoidasida
Apicomplexa
Plasmodium yoelii 6%
Plasmodium vinckei 6%
Plasmodium chabaudi 4%
Plasmodium berghei 3%
Plasmodium (Vinckeia)
Plasmodium malariae 6%
Plasmodium vivax 8%
Plasmodium (Plasmodium)
Plasmodium knowlesi 11%
Plasmodium cynomolgi 11%
4% [other Plasmodium]
3% Plasmodium coatneyi
15% Plasmodium relictum
3% Plasmodium gaboni
14% Plasmodium (Laverania)
Plasmodium
Reads: 71
Unassigned: 3
Rank: genus
86% of Aconoidasida
53% of Apicomplexa
2% of Eukaryota
0.04% of Root

B

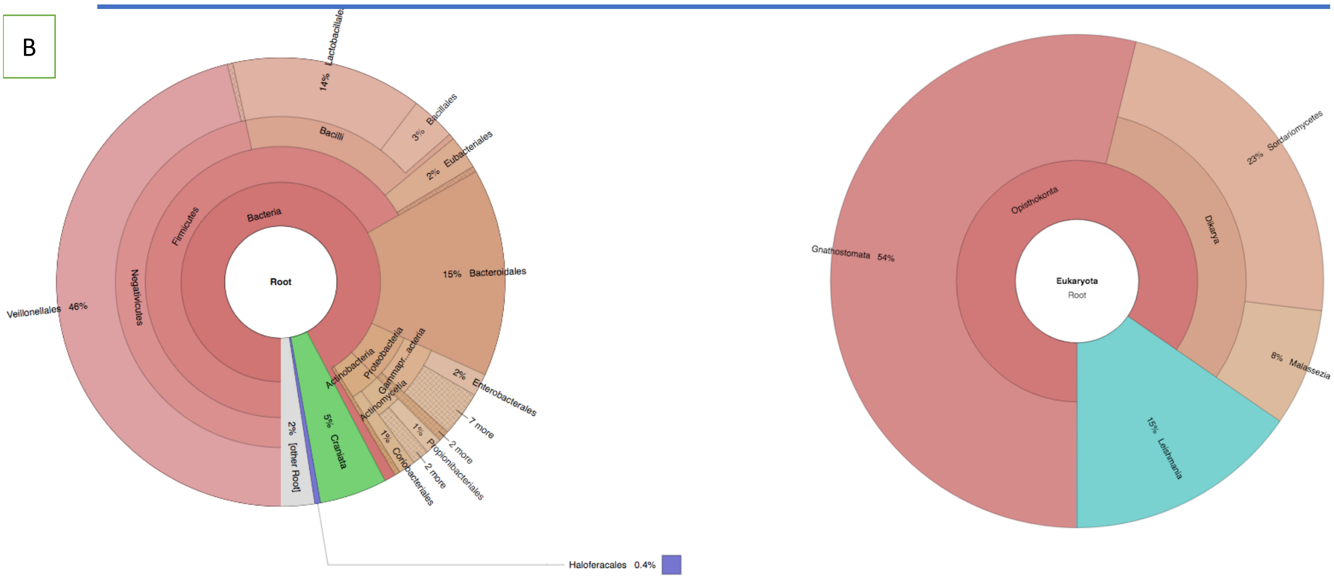

Root
Bacteria
Firmicutes
Negativicutes
Veillonellales 46%
Bacilli
14% Lactobacillales
3% Bacillales
2% Eubacteriales
15% Bacteroidales
Actinobacteria
Proteobacteria
Gammapr...acteria
Actinomycetia
2% Enterobacterales
7 more
1% Propionibacteriales
2 more
1% Coriobacteriales
2 more
5% Craniata
2% [other Root]
Haloferacales 0.4%
Eukaryota
Root
Opisthokonta
Dikarya
Gnathostomata 54%
23% Sordariomycetes
8% Malassezia
15% Leishmania

C

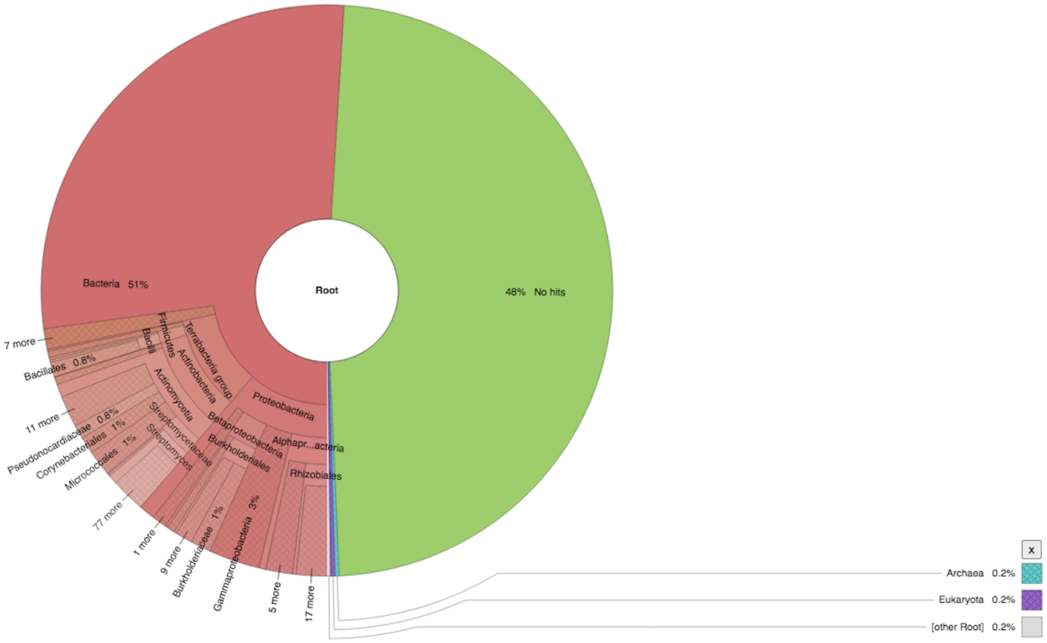

Root
Bacteria 51%
48% No hits
7 more
Bacillales 0.8%
11 more
Pseudonocardiaceae 0.8%
Corynebacteriales 1%
Micrococcales 1%
77 more
1 more
9 more
Burkholderiaceae
Gammaproteobacteria
5 more
17 more
Firmicutes
Bacilli
Terrabacteria group
Actinobacteria
Actinomycetia
Streptomycetales
Streptomycetaceae
Proteobacteria
Betaproteobacteria
Burkholderiales
Alphapr...acteria
Rhizobiales
1%
3%
x
Archaea 0.2%
Eukaryota 0.2%
[other Root] 0.2%
